# A Note regarding Problems with Interaction and Varying Block Sizes in a Comparison of Endotracheal Tubes

**DOI:** 10.1155/2014/956917

**Published:** 2014-07-15

**Authors:** Richard L. Einsporn, Zhenyu Jia

**Affiliations:** ^1^Department of Statistics, The University of Akron, 302 Buchtel Common, Akron, OH 44325-1913, USA; ^2^Department of Family and Community Medicine, Northeast Ohio Medical University, Rootstown, OH 44272, USA

## Abstract

A randomized clinical experiment to compare two types of endotracheal tubes utilized a block design where each of the six participating anesthesiologists performed tube insertions for an equal number of patients for each type of tube. Five anesthesiologists intubated at least three patients with each tube type, but one anesthesiologist intubated only one patient per tube type. Overall, one type of tube outperformed the other on all three effectiveness measures. However, analysis of the data using an interaction model gave conflicting and misleading results, making the tube with the better performance appear to perform worse. This surprising result was caused by the undue influence of the data for the anesthesiologist who intubated only two patients. We therefore urge caution in interpreting results from interaction models with designs containing small blocks.

## 1. Introduction

A clinical research investigation by Radesic et al. [1] compared two types of endotracheal tubes (ETTs) used by anesthesiologists. The original plan for the study utilized a generalized randomized block design [2, 3] (stratified allocation), in which each of six anesthesiology providers (hereafter “APs”) was to use one type of tube for five patients and the other type of tubes for five patients, with assignment of patient to tube being randomized. Three dependent variables obtained for each patient were used to compare the types of tubes: time to complete the intubation, number of times the insertion had to be momentarily stopped and the tube redirected, and a rating by the AP of the difficulty of the insertion. It was anticipated that there could be some interactive effect between the type of tube and the AP with respect to these response variables, in that the differences between the tube types could vary according to the APs' proficiencies and preferences.

In the course of conducting this study, it turned out that some of the APs who were enlisted to participate were seldom available, while others were frequently available. In order to complete the investigation within an allotted time frame, the number of patients per AP was altered with more than ten patients for some APs and fewer than ten for others. Still, each AP had an even number of patients with half being randomized to each type of tube. One particular AP had only two patients, one per tube type. In the original analysis presented in Radesic et al. [1], the researchers deemed this AP to have done too few intubations and excluded that data from the analysis. A further analysis that did include the data for this AP revealed a spurious result that conflicts with the conclusions of the original study. It is this contradictory finding that is the focus of this paper. Such a result should sound a note of caution to data analysts who include interaction terms in their models.

In this paper, we first provide some additional details of both the design and original analysis of the anesthesiology tube study by Radesic et al. Then we will illustrate the specific problem that arises when an interaction term is added to the statistical model. Finally, we discuss how such a problem could arise in many other situations where an interaction term may be included in a model.

## 2. Materials and Methods

The purpose of the study by Radesic et al. [1] was to compare the performance of two types of ETTs when used in conjunction with the GlideScope, a video laryngoscope. The Parker Flex-Tip (PFT) and the standard Mallinckrodt were the two types of ETTs used in this study. The GlideScope allows the AP to visualize the airway structures when passing the ETT into the oral pharynx, through the glottis, and into the trachea.

Six APs and 60 patients participated in the study. In the modified design, one AP intubated 22 patients, another intubated 18 patients, three APs performed 6 intubations each, and one AP only performed two intubations. The APs were balanced with respect to the ETT type, in that half of each AP's intubations were done with the PFT tube and half with the standard Mallinckrodt tube. In the original analysis [1], the data for the AP who did only two intubations was discarded, leaving a sample size of 58 patients, utilizing data for only five APs. The three dependent variables were (1) time for ETT insertion, (2) number of ETT redirections, and (3) ease of use rating by the AP immediately following each intubation. Values for the first two dependent variables were determined precisely by means of viewing a video recording of each intubation. To rate the ease of use, a 100 mm visual analog scale (VAS) was used, with 0 representing “easiest insertion” and 100 representing “hardest insertion.” After each intubation, the AP made a mark along this 100mm line to rate the difficulty of the insertion.

The analysis presented in [1] utilized data for only the 58 patients who were intubated by the 5 APs who did six or more intubations, excluding the AP who did only two intubations. A two-factor ANCOVA model was used, with ETT type and AP being the two designed factors and two patient characteristic variables serving as covariates. These were the Cormack-Lehane view (2 categories) and whether the muscles were paralyzed, as determined by observation of nerve stimulation. The model included interaction terms for the ETT type with each covariate and with the AP factor. The AP was entered into the model as a random effect. Two of the dependent variables were transformed using logs in order to correct for skewness.

When the results were averaged for the 58 patients (aggregated over the five APs and the covariates), the PFT tube had lower (better) mean responses on each of the dependent variables. Likewise, for all three dependent variables, the adjusted means resulting from the model described above were lower for the PFT. *P* values for two of the dependent variables—time to intubate and difficulty rating—were below .01.

In this paper, we will do a similar analysis, this time using the data for all 60 patients and all six APs. To make our point in the most straightforward fashion, our analysis will exclude the two covariates. For the same reason, we will keep the dependent variables in their original units, rather than using log transformations. (The presence of covariates in the model or the use of transformed data does not change the essence of the results.)

## 3. Results and Discussion

Table 1 shows the mean values for each of the dependent variables when the data are aggregated over all six APs. For each dependent variable, the mean response is lower (better) for the PFT tube than for the standard ETT. The results are presented separately for each AP in Table 2. It can be seen that the fourth anesthesiology provider (AP #4) had one patient who was difficult to intubate and one for whom intubation was very easy. Whether this is due to the type of tube or to patient characteristics cannot be sorted out statistically due to confounding.

### 3.1. Additive Model versus Interactive Model Results

First, consider the results of an additive model in which the factors are tube type (fixed) and anesthesiology provider (random). Such a model will allow us to compare the tube types, while adjusting for potential differences among the APs with respect to the dependent variables. For example, some APs could be faster at performing intubations than others. Variation in the dependent variables due to AP differences would then be accounted for and removed from the “error term” used for comparing the tube types. Univariate two-way ANOVAs were run for each of the three dependent variables. According to ANOVA *F*-tests, the difference between the PFT tube and standard tube was not found to be statistically significant for any of the three effectiveness measures. (This is also true if the data for AP #4 are removed.) However, the adjusted mean for the PFT tube was lower (better) than for the standard tube on each of the three dependent variables (Table 3).

In order to allow for the possibility that the differences between the tube types may vary among APs, an interaction term was added to the model. For example, some APs may tend to perform better with one tube while other APs do better with the other tube. Again, univariate ANOVAs were run for each of the three dependent variables, this time with the interaction term, tube type ∗ AP, included in the model. Surprisingly, the adjusted means resulting from these analyses make it appear that the PFT tube performs worse than the standard tube (Table 3). Again, differences are not statistically significant according to the ANOVA *F*-tests.

The adjusted means shown in Table 3 were produced using the General Linear Model ANOVA platform in Minitab V.16 and are the same as those obtained using PROC GLM in SAS V.9.3. To its credit, Minitab's default output flags both of the data points for AP #4 as having “large leverage” for both the additive and interaction models. We also note that the same adjusted means are produced even if the APs are entered into the model as fixed rather than random effects.

We believe that the results obtained using the interaction model are misleading due to the undue influence of the results for the one AP who intubated only one patient with each type of ETT. Further, we were somewhat surprised by this, because the design was balanced in the sense that each AP used each ETT type the same number of times, meaning that the ETT and AP factors are orthogonal in the design matrix.

### 3.2. Illustration

The misleading results obtained in the ETT study could arise in many similar situations. Here is a simple example to illustrate the problem in the context of a two-factor factorial analysis. Suppose that Factors A and B have *a* and *b* levels, respectively, and that, within each level of Factor A, the same number of observations is obtained for each level of B, although this number may vary among the levels of A. As in the anesthesia tube study, we are primarily focusing on the impact of only one factor, here, Factor B.

If *n*_*ij*_ represents the number of observations for the *i*th level of A and *j*th level of B, then *n*_*i*1_ = *n*_*i*2_ = ⋯ = *n*_*ib*_ for *i* = 1,2, …, *a*. Consider the case where *a* = 3 and *b* = 2; *n*_1*j*_ = 5, *n*_2*j*_ = 4, and *n*_3*j*_ = 1, *j* = 1,2. Suppose the values of the dependent variable are as shown in Table 4.

In this case, the raw means for the two levels of Factor B differ by 3.0 with the B1 mean higher than the B2 mean (Table 5). Using an additive model, PROC GLM in SAS produces adjusted means that are also 3.0 units apart, and the difference is statistically significant (*P* = .030). For the interaction model, the adjusted means for the two levels of B are reversed in order of magnitude, though the difference is not statistically significant (*P* = .367 using SAS type III sums of squares).

Minitab's General Linear Model ANOVA produces the same results for both the additive and interaction models. To its credit, Minitab also issues a warning in its output that the two observations for A3 have high leverage. To investigate this further, we performed regression analyses, which allowed us to assess the leverage and influence of the two data values for A3. To do this, we created indicator variables for A1, A2, and B1 and multiplicative interaction terms A1∗B1 and A2∗B1. Then we ran a regression analysis with both an additive model (Y versus A1, A2, and B1) and an interaction model (Y versus A1, A2, B1, A1∗B1, and A2∗B1), requesting that influence diagnostics be included in the output (INFLUENCE option in PROC REG). For the additive model, each of the two A3 observations had somewhat high leverage (hat diagonal = .55) and strong influence on the estimate for B1 (DFBETAS = −3.11) (see Belsley et al. [4]). However, for the interaction model, these two points had the maximum possible leverage (hat diagonals = 1.00) and extreme influence on all the coefficient estimates (DFBETAS all infinite/undefined). With only one observation at each combination of A3 and B, it is clear that an interactive model will fit the response variable exactly and thus the maximum leverage.

## 4. Conclusion

There are many clinical studies, such as the ETT comparison described here, where allocation of patients to treatments may be blocked or stratified (see [5] for a discussion of stratification in the clinical trial setting). For example, patients may be stratified by center, race, or disease status. In such cases, additive models for comparing the treatments will properly adjust for net differences in the dependent variables for the different strata. However, it may make sense for an interaction model to be used in the model as well. For example, the benefit afforded by one treatment over another may be greater for one racial group than for another. If one or more of the strata are very small in size, then the phenomenon illustrated by the examples of this paper suggests caution be used in interpretation of the results. Data for the small strata may have undue influence on the findings, since these observations will have high leverage. As shown here, this problem holds even for the “unbiased” case where, within any stratum, an equal number of subjects receive each treatment. In light of these observations, we recommend that strata or blocks of size two be omitted from the data if an interaction model is used. This advice was followed in the original ETT comparison analysis [1], where the AP who intubated only two patients was removed from the data.

## Figures and Tables

**Table 1:** Intubation outcomes for the Parker Flex-Tip and standard tubes.

Dependent variables	Parker Flex-Tip (*n* = 30) mean (SD)	Standard ETT (*n* = 30) mean (SD)
Time for ETT insertion (sec)	10.9 (7.5)	12.4 (7.3)
Number of redirections	0.7 (1.5)	1.3 (2.7)
Difficulty of insertion rating	14.3 (14.9)	17.4 (19.7)

**Table 2:** Mean intubation outcomes for the Parker Flex-Tip and standard tubes for each of the six anesthesiology providers.

Dependent variables	Parker Flex-Tip	Standard ETT
AP#1	*N* = 3	*N* = 3
Time for ETT insertion (sec)	9.0	14.0
Number of redirections	1.0	2.0
Difficulty of insertion rating	16.7	19.0
AP#2	*N* = 11	*N* = 11
Time for ETT insertion (sec)	6.7	9.9
Number of redirections	0.0	0.7
Difficulty of insertion rating	3.7	11.5
AP#3	*N* = 9	*N* = 9
Time for ETT insertion (sec)	14.7	17.1
Number of redirections	1.6	2.4
Difficulty of insertion rating	21.7	31.8
AP#4	*N* = 1	*N* = 1
Time for ETT insertion (sec)	15.0	5.0
Number of redirections	3.0	0.0
Difficulty of insertion rating	60.0	7.0
AP#5	*N* = 3	*N* = 3
Time for ETT insertion (sec)	8.0	6.0
Number of redirections	1.0	0.0
Difficulty of insertion rating	13.3	11.7
AP#6	*N* = 3	*N* = 3
Time for ETT insertion (sec)	18.7	14.7
Number of redirections	0.0	0.7
Difficulty of insertion rating	14.2	3.0

**Table 3:** Least squares adjusted means for each type of tube using an additive model or an interaction model.

Dependent variables	Additive model		Interaction model	
	Parker Flex-Tip	Standard ETT	Parker Flex-Tip	Standard ETT
Time for ETT insertion (sec)	10.8	12.3	12.0	11.1
Number of redirections	0.8	2.3	1.1	1.0
Difficulty of insertion rating	16.3	19.4	21.6	14.0

*Note.* The adjusted means were obtained using the General Linear Model ANOVA platform in Minitab V.16 and are the same as those obtained using PROC GLM in SAS V.9.3.

**Table 4:** Hypothetical data for a two-factor study.

	Factor B level 1	Factor B level 2
Factor A level 1	10 11 12 11 10	8 6 5 7 7
Factor A level 2	9 11 12 10	6 7 4 5
Factor A level 3	4	15

**Table 5:** Raw and adjusted means for the two levels of B for the hypothetical data.

	Factor B level 1	Factor B level 2
Raw means	10.00	7.00
Adjusted means; additive model	10.23	7.23
Adjusted means; interaction model	8.43	9.03
